# Supplementary material for: Robust V(D)J Recombination in the Absence of SA1/2-mediated Chromatin Loops
Source: bioRxiv. 2026 Oct 11:2026.10.10.757971. Preprint. [Version 1] doi: 10.64898/2026.10.10.757971 (PMC13692300; doi:10.64898/2026.10.10.757971)

Supplementary Figure 4 | TdT gene expression and protein level in 129SV-background *v-Abl* cells. **a**, TdT gene (*dntt*) expression level shown in Gro-seq data in G0/G1-arrested bone marrow pro-B cell, G0/G1-arrested 129SV-*v-Abl* parental cells, and G0/G1-arrested 129SV-*v-Abl* *SA1$^{-/-}$*/SA2-depleted cells. **b**, Western blotting analysis of TdT protein level in 129SV-background *v-Abl* cell lines: parental, SA1-depleted, SA2-depleted, and *SA1$^{-/-}$*/SA2-depleted cells. Total thymocytes cells were used as positive TdT protein control. Antibodies are indicated on the left, and protein sizes are indicated on the right.

Supplementary Figure 4

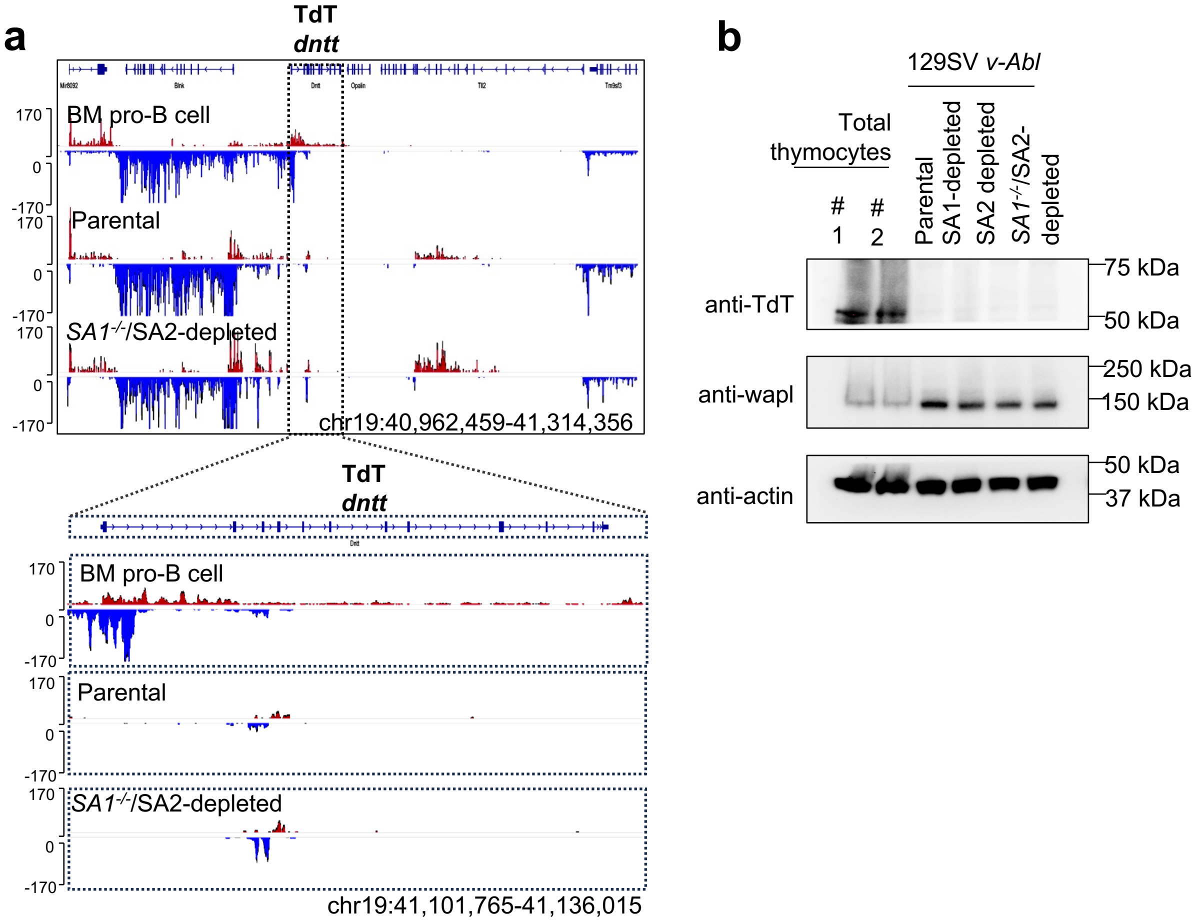

Supplement: Supplementary file 1 [file media-1.zip › Supplementary information 4.pdf]
